# Membrane-Mediated Action of Phosphodiesterase 5 Inhibitors

**DOI:** 10.3390/pharmaceutics17050563

**Published:** 2025-04-24

**Authors:** Anna I. Malykhina, Svetlana S. Efimova, Olga S. Ostroumova

**Affiliations:** Laboratory of Membrane and Ion Channel Modeling, Institute of Cytology of Russian Academy of Sciences, Tikhoretsky Ave. 4, St. Petersburg 194064, Russia

**Keywords:** PDE inhibitors, sildenafil, vardenafil, tadalafil, lipid interdigitation, lipid bilayer, membrane permeability

## Abstract

**Background/Objectives**: Phosphodiesterase 5 (PDE5) inhibitors, sildenafil, vardenafil, and tadalafil, activate the cyclic guanosine monophosphate pathway resulting in vascular smooth muscle relaxation. They have been tested for a broad variety of conditions from cancer to Alzheimer’s disease with a positive impact. The known mechanism of action of these drugs could not explain such a plethora of effects. We studied the influence of PDE5 inhibitors on lipid bilayers as a possible application point of their action. **Methods**: To monitor the membrane changes induced by PDE5 inhibitors, the differential scanning microcalorimetry and the molecular dynamics simulation were used. **Results**: We found that sildenafil, vardenafil, and tadalafil change elastic properties of model membranes: PDE5 inhibitors disorder thin membranes and order thick membranes. Moreover, PDE inhibitors were able to induce lipid interdigitation. To address the biological aspect of the findings, we performed molecular dynamics on smooth muscle cell’s lipid raft treated with PDE5 inhibitors and revealed the increased density of the lipids. Furthermore, we showed that the lipid condensation in the PDE inhibitors presence increases nitric oxide permeability. **Conclusions**: The obtained results may be of biological relevance as lipid raft thickening might have an impact on membrane protein function. Moreover, improved nitric oxide flow through membrane may partially explain therapeutic action of these drugs. The presented results are useful for finding novel implications for PDE inhibitors.

## 1. Introduction

Phosphodiesterase 5 (PDE5) is an enzyme that degrades the phosphodiester bond in a key cellular second messenger, cyclic guanosine monophosphate (cGMP), converting it to an inactive form—5′-GMP. PDE5 is broadly expressed in the vascular and visceral smooth muscle cell, including corpus cavernosum and cardiovascular system, where it regulates vascular tone [1]. The FDA approved PDE5 inhibitors, sildenafil, tadalafil, and vardenafil, are registered for the treatment of erectile dysfunction, pulmonary arterial hypertension and lower urinary tract symptoms. Besides that, PDE5 inhibitors are being extensively tested in an application to a broad spectrum of different diseases such as myocardial infarction, cancer, diabetes, Alzheimer’s disease and others [2]. The computational method is widely applied for the search of new inhibitors for PDE5 or new targets of existing drugs [3,4,5]. Both strategies imply only the protein target for PDE5 inhibitors.

**Figure 1:**
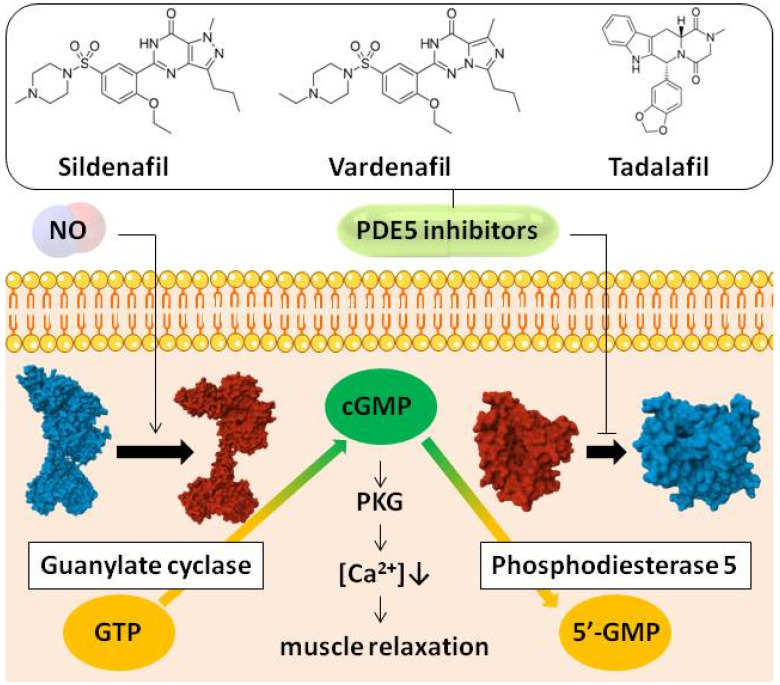
Scheme of PDE5 inhibitors mechanism of action. Nitric oxide (NO) diffuses into cell and activates guanylate cyclase, which converts guanosine triphosphate (GTP) to cyclic guanosine monophosphate (cGMP). cGMP is the second messenger that initiates a cascade through protein kinase G (PKG) activation and calcium level degrease ([Ca^2+^]↓), resulting in muscle relaxation. cGMP level is controlled by phosphodiesterase 5 (PDE5), which converts it to inactive 5′-guanosine monophosphate (5′-GMP). PDE5 inhibitors block this enzyme, promote cGMP accumulation, and stimulate muscle relaxation. Guanylate cyclase and phosphodiesterase 5 images in an active (red) and inactive form (blue) are taken from PDB database (guanylate cyclase active—6JT2, inactive—6JT0, phosphodiesterase 5 active—2H40, inactive—2H42).

The main clinical action of PDE5 inhibitors is a smooth muscle relaxation, which is accomplished through cGMP accumulation (Figure 1). The process begins with nitric oxide (NO) diffusion into a muscle cell from noncholinergic, nonadrenergic neurons or endothelial cells where it is synthesized under certain stimulation. NO activates guanylate cyclase, which converts guanosine triphosphate (GTP) to cGMP. The PDE5 enzhyme maintains cGMP level by cGMP degradation. Thus, PDE5 inhibition leads to cGMP increase, which initiates a cascade through protein kinase G (PKG) activation and calcium level decrease leading to pronounced muscle relaxation.

Apart from this well-established mechanism of action, there are some reports that describe the increase in permeability under PDE5 inhibitors treatment. For example, sildenafil and vardenafil doubled the level of transport of radioactive sucrose into the brain tumor in rat models as compared to the untreated controls [6]. The cellular uptake of anticancer drugs doxorubicin, carboplatin, dextran, and trastuzumab in H1915 cells exhibited almost a twofold increase in vardenafil presence [7]. Tadalafil was shown to enhance rituximab treatment efficacy by improving the microvascular permeability in mice brain lymphoma [8].

One explanation of the PDE5 inhibitors-induced permeability may be the activation of caveolae-mediated endocytosis as caveolae pathway inhibitors brought chemotherapeutic drug uptake down to control levels [9]. It has now been shown that caveolae can flatten in response to a membrane stretch leading to the caveolin–cavin module dissociation [10]. Caveolin 1 is phosphorylated in response to several mechanical stimuli, which was linked to extracellular matrix remodeling and caveolar endocytosis [11]. Thus, mechanical properties of the membrane are important for PDE5 inhibitors-associated drug permeability. It is known that PDE5 inhibitors change physicochemical properties of the membranes, including the alteration in the boundary and dipole potential bilayers [12]. This work investigates the influence of PDE5 inhibitors on elastic properties of membranes using the differential scanning microcalorimetry and the molecular dynamics simulation. The combination of calorimetry, which evaluates the macroscopic thermodynamic parameters of the system, and molecular dynamics, which describes the processes at the molecular level, should allow one to characterize the action of inhibitors on model lipid membranes in detail.

## 2. Materials and Methods

### 2.1. Materials

Synthetic 1,2-dimyristoyl-*sn*-glycero-3-phosphocholine (DMPC, 14:0 PC, lipid with two 14-carbon saturated acyl chains at the *sn*-1 and *sn*-2 positions of glycerophosphocholine), 1,2-dipalmitoyl-*sn*-glycero-3-phosphocholine (DPPC, 16:0 PC, lipid with two 16-carbon saturated acyl chains at the *sn*-1 and *sn*-2 positions of glycerophosphocholine), 1,2-distearoyl-*sn*-glycero-3-phosphocholine (DSPC, 18:0 PC, lipid with two 18-carbon saturated acyl chains at the *sn*-1 and *sn*-2 positions of glycerophosphocholine), and 1,2-diarachidoyl-*sn*-glycero-3-phosphocholine (DAPC, 20:0 PC, lipid with two 20-carbon saturated acyl chains at the *sn*-1 and *sn*-2 positions of glycerophosphocholine) were obtained from Avanti Research (Avanti Research, Inc., Alabaster, AL, USA). Sildenafil, vardenafil, and tadalafil were purchased from Sigma-Aldrich Company Ltd. (Gillingham, UK). The chemical structures of the tested agent are presented in Figure 1.

### 2.2. Differential Scanning Microcalorimetry (DSC)

Giant unilamellar vesicles were prepared from the liposome suspension contained 5 mM pure DMPC, DPPC, DSPC, and DAPC and buffer solution (5 mM HEPES-KOH at pH 7.4) by the electroformation method (standard protocol, 3 V, 10 Hz, 1 h, 35 °C (DMPC), 55 °C (DPPC), 65 °C (DSPC), and 75 °C (DAPC)). The tested sildenafil, vardenafil, and tadalafil were added to aliquots to obtain a molar ratio of 100:1, 50:1, 25:1, 10:1 and 5:1. The liposomal suspension was heated and cooled at a constant rate of 0.2 and 0.3 °C/min, respectively. The reversibility of the thermal transitions was assessed by reheating the sample immediately after the cooling step from the previous scan. The temperature dependence of the excess heat capacity was analyzed using Calisto Processing (Setaram, Caluire-et-Cuire, France). The peaks on the thermograms were characterized by the maximum temperature of the main phase transition (*T_m_*) of DMPC, DPPC, DSPC, and DAPC, the width of the main peak, Δ*T_b_*, characterizing the inverse cooperativity of the melting, the enthalpy of the main phase transition (an area of the main peak, ∆*H*), and the difference in the transition temperatures between heating and cooling scans (∆*T_h_*).

The values of Δ*T_m_*, ΔΔ*T_b_*, ΔΔ*T_h_*, and ΔΔ*H* were presented from individual experiments.

### 2.3. Molecular Dynamics Simulation (MD)

Topology parameters of PDE5 inhibitors (sildenafil, vardenafil and tadalafil) were based on known 3D structures [13] and were generated using CGenFF [14]. Parameters for NO, in particular, the van der Waals parameters and Morse potential parameters that describe N-O bond were taken from [15]. GROMACS 2023.2 [16] was used to perform MD simulation with CHARMM36m all-atom force field [17].

Model membranes were assembled in CHARMM-GUI Membrane Builder [18] and contained 120 lipid molecules of DMPC, DPPC or DSPC. Model membrane that imitates caveolin-1-associated raft of smooth muscle cell plasma membrane consisted of 6 1-palmitoyl-2-oleoyl-inositol (POPI), 12 3-palmitoyl-2-oleoyl-D-glycero-1-phosphatidylserine (POPS), 46 3-palmitoyl-2-oleoyl-D-glycero-1-phosphatidylcholine (POPC), 32 3-palmitoyl-2-oleoyl-D-glycero-1-phosphatidylethanolamine (POPE), 22 sphingomyelin (18:1) (SSM) and 82 cholesterol molecules according to experimental data [19]. PDE5 inhibitors were inserted in the headgroups region of membrane in lipid:drug ration 10:1. 10 NO molecules were added to water in a raft-membrane simulation. Energy minimization with the steepest descent algorithm was followed by a six-step equilibration process with gradually turning off the position restraints on lipid molecules (default parameters generated by CHARMM-GUI). Production simulations were performed for 100 ns. Simulations for differential scanning microcalorimetry comparison were conducted at the constant temperature of 25 °C (at this temperature DPPC, DSPC and DAPC are in gel phase), and simulations for raft-membrane permeability were conducted at 37 °C using V-rescale thermostat; the pressure in all simulations was 1 bar employing semi-isotropic pressure coupling approach with a C-rescale barostat [20,21]. The time constant of coupling for temperature and pressure was 1 and 5 ps, respectively. The Particle Mesh Ewald method was employed to treat long-range electrostatic interaction with a short-range cutoff 1.2 nm [22], while the shifted Lennard–Jones potential algorithm was used to calculate the van der Waals interactions with a general cutoff of 1.2 nm and a shifting cutoff of 1.0 nm. The time-step of the MD simulations was 0.002 ps. The trajectory in production simulations was recorded every 10 ps. Convergence was assessed using RMSD analysis (Supplementary Figure S1).

Visualization was conducted on VMD [23]. Area per lipid (APL), membrane thickness and lipid interdigitation were estimated by MEMBPLUGIN [24]. Lipid interdigitation was defined as a fraction of mass overlap. *D_i_*_nt_ is a ratio between lipid interdigitation in the presence and absence of modifying agent. NO diffusion was assessed using gmx msd tool. All parameters were averaged for the interval from 20 ns till the end of the simulation and presented as a mean ± standard deviation. The diffusion coefficient was determined by the linear regression of the MSD (mean squared displacement) graph on its linear section, which was 2–10 ns long depending on simulation. Partition coefficient (*K_p_*) was determined as a ration between NO molecules in a hydrophobic core and in a hydrophilic part of simulation box, which included the headgroups region and water. Diffusion in membrane (*D_m_*) was calculated as ration of diffusion coefficient in *z* axis and in *xy* plane of the simulation box according to [25]. Permeability of NO was assessed by simplified formula $P_{m}=D_{m}\cdot K_{p}/\delta$, where δ is the thickness of the membrane.

## 3. Results and Discussion

### 3.1. PDE Inhibitors Change Elastic Properties of Model Membranes

**Figure 2:**
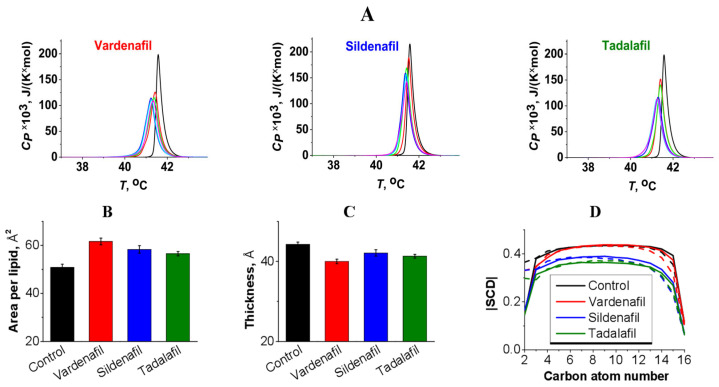
(**A**) Heating thermograms of DPPC melting in the absence (control, *black curves*) and presence of sildenafil, vardenafil, and tadalafil at different molar ratio of 100:1 (*red curves*), 50:1 (*green curves*), 25:1 (*blue curves*), 10:1 (*cyan curves*) and 5:1 (*purple curves*). (**B**) area per lipid, (**C**) membrane thickness and (**D**) order parameter for the acyl tails of DPPC membrane in the absence (control, *black*) and presence of vardenafil (*red*), sildenafil (*blue*) and tadalafil (*green*) at lipid:drug ratio of 10:1 based on MD data at 25 °C. For |SCD| *sn*-1 and *sn*-2 acyl chains marked as solid and dashed line respectively.

We studied the effect of sildenafil, vardenafil, and tadalafil on the thermotropic behavior of the neutral DPPC using differential scanning calorimetry. Figure 2A demonstrates the heating thermograms of DPPC in the absence (control, *black curve*) and presence of PDE5 inhibitors at different molar ratio of 100:1 (*red curves*), 50:1 (*blue curves*), 25:1 (*green curves*), 10:1 (*cyan curves*) and 5:1 (*purple curves*). Table 1 demonstrates that sildenafil, vardenafil, and tadalafil were characterized by a slight effect on the main thermothropic parameters of the DPPC, indicating an interaction of the small molecules with bilayer and their minor disordering action. Sildenafil and tadalafil did not practically affect the main thermothropic parameters of the DPPC at different ratio to lipid (Δ*T_m_*, ΔΔ*T_b_*, and ΔΔ*T_h_* was about 0.2 °C), confirming the slight interaction between small molecules and PC. The biphasic dependence of DPPC transition temperature (*T*_m_) on vardenafil concentration was observed: an addition of vardenafil up to ratio 25:1 led to a decrease in *T*_m_ and a subsequent increase in vardenafil concentration up to 5:1 caused an enlargement in *T*_m_-value (Figure 2A, Table 1). At the same time, Table 1 shows that the changes in the half-width of the DPPC transition peak, the difference in the *T*_m_-values between heating and cooling scans (*T*_m_-hysteresis), and the transition enthalpy (Δ*H*) increased monotonically with increasing vardenafil concentration. The observed biphasic behavior of *T*_m_-value in the presence of vardenafil might indicate that it induces quasi-interdigitated phase when the hydrophobic tails of the phospholipids from the opposite membrane leaflets interlock and interpenetrate leading to a decrease in the membrane thickness and an increase in the membrane packing density [26].

We also assessed PDE inhibitors influence on an area per lipid (APL), membrane thickness and lipid tails order parameter (SCD) using the molecular dynamics. SCD reflects the orientational mobility of each C-H bond along the aliphatic lipid tails and membrane fluidity. A total of 12 drug molecules were initially embedded to a solvated lipid bilayer system composed of DPPC, which is equal to the experimental molar lipid:drug ratio of 10:1. Simulation was performed for 100 ns at 25 °C. Vardenafil explicitly increased APL by 10.9 Å^2^ comparing to sildenafil and tadalafil (7.6 and 5.7 Å^2^, respectively) (Figure 2B). Membrane thickness decrease was also profound at vardenafil addition (4.3 Å) followed by tadalafil (3.0 Å) and sildenafil (2.2 Å) (Figure 2C). These data provide evidence on the disordering action of PDE inhibitors and exhibit vardenafil as the most potent disordering agent. However, on the SCD graph one could notice that the order of lipid tails practically did not change after vardenafil addition comparing to the control (Figure 2D). This inconsistency also implicitly indicates the presence of lipid quasi-interdigitation caused by increasing head–head repulsion, which increases APL and reduces membrane thickness without diminishing the order of lipid tails [27]. The interdigitated structure is thought to be induced by the enlargement in the strength of the repulsive interaction between the lipid headgroups due to intercalation of the agent into bilayer and subsequent expanding in the effective volume of the lipid headgroups leading to the increase in the mismatch between effective headgroup and tail sizes. Such interaction leads to an increase in lipid heads hydratation. Indeed, the mean number of hydrogen bonds between DPPC and water increased by 5% in vardenafil presence.

**Figure 3:**
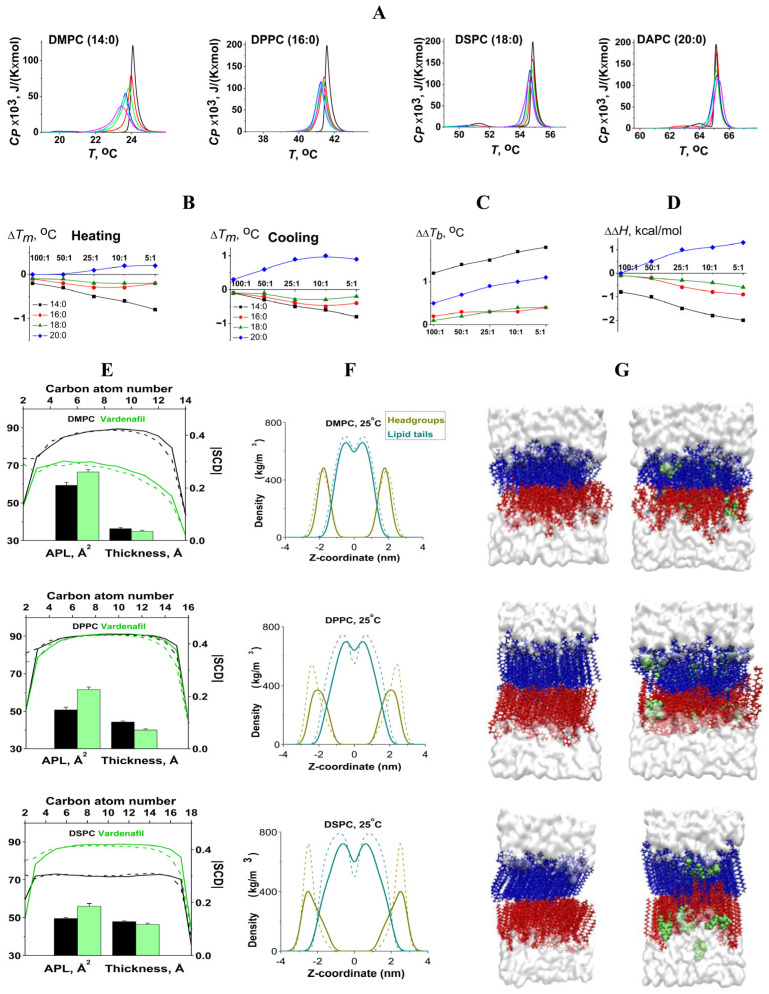
(**A**) Heating thermograms of DMPC, DPPC, DSPC, and DAPC melting in the absence (control, *black curves*) and presence of vardenafil at different molar ratio of 100:1 (*red curves*), 50:1 (*green curves*), 25:1 (*blue curves*), 10:1 (*cyan curves*), and 5:1 (*purple curves*). (**B**–**D**) The dependences of the parameters characterizing the thermotropic behavior of DMPC (14:0, *black curves*), DPPC (16:0, *red curves*), DSPC (18:0, *green curves*), and DAPC (20:0, *blue curves*) on lipid:vardenafil ratio: (**B**) the changes in melting temperature (∆*T_m_*) at heating (*left panel*) and cooling scans (*right panel*), (**C**) the width of the main peak (∆∆*T_b_*), and (**D**) the transition enthalpy (∆Δ*H*). (**E**) The influence on area per lipid molecule (APL), membrane thickness, and order parameter for the acyl tails (SCD) in lipid bilayers of different composition in the absence (control, *black*) and presence of vardenafil (*green*). For |SCD| *sn*-1 and *sn*-2 acyl chains marked as solid and dashed line, respectively. (**F**) Density plot (*left-side panel*) of headgroups region (*dark yellow*) and lipid tails (*dark cyan*) of control membranes (*dashed line*) and membranes with vardenafil (*solid line*) consisted of DMPC (*upper panel*), DPPC (*middle panel*), DSPC (*lower panel*), and (**G**) visualization of last simulation step of these membranes (*right-side panel*) based on MD data at 25 °C at lipid: vardenafil ratio of 10:1. On visualization upper and lower leaflet of membranes colored in blue and red accordingly for better interdigitation observation, water presented in white, vardenafil—in green.

Interdigitation of lipids can be induced by a number of agents such as ethanol, benzyl alcohol, vinblastine, vinorelbine, atropine, tetracaine, labdanes, chlorpromazine, etc. [28]. However, for PDE5 inhibitors it was not previously shown. According to the literature data, the propensity of saturated PC to interdigitate under chemical inducers enhances as the hydrocarbon chain length increases [29]. To check if the interdigitation actually took place, we further investigated vardenafil as it showed marked results both on DCS and MD. We varied the membrane thickness using the lipid with shorter tails DMPC (14:0) and the lipids with longer chains DSPC (18:0) and DAPC (20:0) compared to DPPC (16:0). Figure 3A shows the endotherms of phase transitions of phosphocholines with saturated hydrocarbon chains of different lengths in the absence (*control*) and presence of vardenafil at different molar lipid ratios. Vardenafil is characterized by a pronounced disordering effect on the thermotropic behavior of DMPC, the slight effect on the DSPC melting, and the ordering effect on the DAPC (Figure 3A). Figure 3B–D demonstrates the changes in the main thermotropic parameters of the phosphocholines at different lipid:drug ratio. Vardenafil caused the disappearance of the pre-transition peak of all tested phosphocholines from the gel state to the ripple phase independently of the thickness of the phosphocholine membranes. The results presented in Figure 3B are in good agreement with the expected difference in the threshold concentrations of vardenafil that cause the interdigitation of membranes composed of lipids with different tail lengths. This concentration, which means an increase in the repulsion of lipid heads to a value that can no longer be compensated by disordering and tilting of the acyl chains, will entail interdigitation of the chains. It should be expectedly greater for lipids with shorter tails. The disordering effect of vardenafil on the thinnest membranes made of DMPC (14:0) at all tested concentrations, including the lipid:drug ratio of 5:1, indicates the impossibility of the PDE5 inhibitor to interdigitate this bilayer. In the case of medium-length acyl-chain lipid bilayers composed of DPPC (16:0) and DSPC (18:0), the threshold concentration should be about the local extremum of the dependence of Δ*T_m_* on the lipid:vardenafil ratio, i.e., correspond to a ratio of about 10:1 and 25:1, respectively (Figure 3B, cooling). Taking into account the condensing effect of vardenafil on DAPC membranes, a length of 20 hydrocarbon units is probably sufficient for vardenafil to induce interdigitation at the lowest concentrations tested, corresponding to a ratio of less than 100:1. We also assessed vardenafil-induced interdigitation, changes in APL, bilayer thickness, and lipid tails order parameter (SCD) in the membranes composed of lipids with different acyl chains using the molecular dynamics. Figure 3E demonstrates that the APL increased and that the membrane thickness decreased in the presence of vardenafil at lipid:drug ratio of 10:1 compared to control lipid membranes by 7.1 Å^2^, 10.8 Å^2^ and 6.7 Å^2^ and 1.5 Å, 4.3 Å and 1.4 Å for DMPC, DPPC and DSPC, respectively. The lipid tails order dramatically changed under the action of vardenafil exhibiting its disordering effect on DMPC, almost no effect on DPPC and ordering effect on DSPC (Figure 3E). The additional analysis of the MD density plot (Figure 3F,G) showed that the density in the area of the headgroups decreased sharply in the DPPC and DSPC membranes, indicating an increase in head–head repulsion reflected in the growth of APL, while the density in the center of the membrane distinctly increased, which might indicate tail interdigitation. Lipid interdigitation parameter (D_int_) consistently increased in series DMPC < DPPC < DSPC and was equal to 0.86, 1.59 and 2.40, respectively. Thus, these data were in agreement to the DSC results.

Thereby, we found out that PDE inhibitors acted differently depending on membrane thickness: they disordered thin membranes and ordered thick membranes displaying lipid interdigitation. This finding may be biologically relevant as cell membranes contain many lipid rafts with functionally active proteins and channels that are sensitive to membrane thickness and other elastic properties. Another point is the passive diffusion of small molecules that might be influenced by lipid density change.

### 3.2. PDE Inhibitors Affect Elastic Properties of Lipid Rafts and NO Diffusion Coefficient

To study PDE inhibitors action on lipid rafts by MD, we built a bilayer with the composition of caveolin-1-associated raft of smooth muscle cell plasma membrane [19] as the smooth muscle cell is the main target of these drugs. Another important actor of the cGMP pathway is the nitric oxide. Thus, we decided to assess its diffusion by putting 10 NO molecules above a raft-like bilayer composed of POPI/POPS/POPC/POPE/SSM/cholesterol (6/12/46/32/22/82). The 100 ns simulations were conducted at the physiological temperature (37 °C) for the control membrane and the membranes where PDE inhibitors were inserted prior simulation at lipid:drug ratio of 10:1. The changes of MD parameters are presented in Table 2. The APL increase in the presence of vardenafil, sildenafil, and tadalafil by 4.2 Å^2^, 6.1 Å^2^, 2.3 Å^2^ was minor compared to pure DPPC membranes, while membrane thickness decreased significantly by 2.4 Å, 2.2 Å, 1.4 Å for vardenafil, sildenafil and tadalafil, respectively (Table 2). D_int_ was 1.53 for sildenafil and vardenafil and 1.33 for tadalafil, which resembles the interdigitation in DPPC membrane. Such changes in the lipid raft may impede the proper work of membrane proteins. For example, it is known that PDE inhibitors reduced chemotherapeutic drug resistance by inhibiting ABCB1-mediated drug efflux [30]. ATP-binding cassette (ABC) transporters are located in the membrane and overexpressed in multi-drug resistance cancers. It was shown that sildenafil and vardenafil but not tadalafil inhibited ATP-hydrolysis and substrate binding of this transmembrane enzyme without influence on its expression or localization [30,31]. The authors implied that there was a direct interaction between ABCB1 and the inhibitor; however, such interaction is difficult to prove for a transmembrane protein, and the influence of the drug through membrane modification could not be ruled out. It is worth mentioning that, in our MD data, tadalafil had the smallest effect on the physical properties of the lipid raft, which correlates with the poor inhibition of an ABC-transporter by this drug.

**Figure 4:**
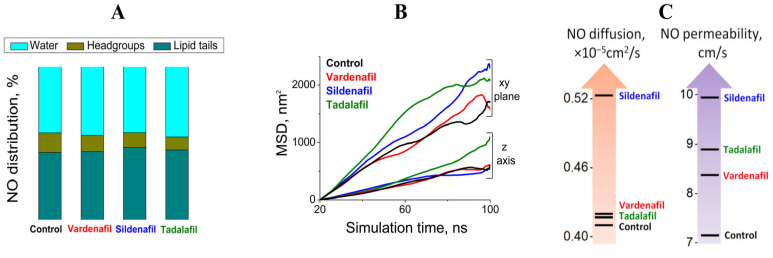
Assessment of NO diffusion in raft-like membrane by MD simulation. (**A**) NO distribution in different region of simulation box: water (light blue), headgroups (dark yellow), and lipid tails (dark cyan) in the control membrane and with PDE inhibitors. (**B**) MSD of NO molecule during MD simulation in the *z*-direction and *xy* plane. (**C**) Comparison of NO diffusion and permeability coefficients in the presence of PDE inhibitors.

The nitric oxide is a small slightly hydrophobic molecule that diffuses across membranes very rapidly due to its favorable partition into membranes and high diffusion coefficient in lipids [32]. For proper analysis of NO permeability, one should consider a different region of the membrane separately. It was shown that in membranes from red blood cells, NO diffusion in the acyl region was 3 times higher than in the headgroup region [33]. Another study revealed that NO diffusion in the headgroup region of phosphatidylcholine membranes is similar to the diffusion in the water, while it increases in acyl-chain area and significantly increases after cholesterol addition [34]. The assessment of NO distribution in our MD simulations exhibited a growth of NO concentration in lipid tails region in membranes with PDE inhibitors compared to the control one and its reduction in the headgroups area indicating an increment of the partition coefficient (*K_p_*) (Figure 4A). The diffusion coefficient in the membrane can be calculated from MSD of NO molecules. However, the setup of simulation assumes the presences of water that baths the membrane making NO MSD not useful for lipid diffusion assessment. To overcome this obstacle, we applied an approach proposed in [25]: to take the ratio of diffusion coefficients in the *z*-direction (perpendicular to the membrane surface) to diffusion coefficient in the *xy* plane (parallel to membrane) (Figure 4B). For control condition, the diffusion coefficient (*D_m_*) was 0.41 × 10^−5^ cm^2^/s, which is in good agreement with the literature MD value in phosphatidylcholine/phosphatidyletanolamine (7/3) membrane (0.35 × 10^−5^ cm^2^/s) and experimental data for the red blood cell membrane (0.5–1.3 × 10^−5^ cm^2^/s) [32,35]. The *D_m_* increases only by 2% with vardenafil and tadalafil, and by 28% with sildenafil (Figure 4C). To evaluate NO permeability, we used a simplified formula from the Methods section and received that *P_m_* was 7.2 cm/s for the lipid raft in the absence of PDE5 inhibitors and 8.4 cm/s, 8.9 cm/s, and 9.9 cm/s in the presence of vardenafil, tadalafil, and sildenafil, respectively (Figure 4C). Experimental data have measured the *P_m_* for red blood cell membranes as 18 cm/s [32]. Lower values in MD simulations might be explained by focusing only on distinct membrane domains. The values might be even higher for biological membranes resulting in a physiological effect. For instance, it was shown that treatment with sildenafil improved the sensitivity of platelets to NO, which prevented platelet aggregation [2].

## 4. Conclusions

PDE inhibitors, sildenafil, vardenafil, and tadalafil, change elastic properties of model membranes depending on their thickness: the drugs disorder thin membranes and order thick membranes. For the first time, we showed with molecular dynamics simulation and differential scanning microcalorimetry that PDE inhibitors are able to induce lipid interdigitation. The obtained results may be of biological relevance as the increased density of the lipid raft revealed on MD might have an impact on a membrane protein function. Moreover, lipid condensation under PDE inhibitors presence improves nitric oxide permeability, which may partially explain therapeutic action of these drugs. The presented results are useful for finding novel indications for PDE inhibitors.

## Figures and Tables

**Table 1:** Thermotropic DPPC characteristics in the presence of vardenafil, sildenafil, and tadalafil.

PDE Inhibitors	Ratio	Δ*T_m_*, °C	ΔΔ*T_b_*, °C	ΔΔ*T_h_*, °C	ΔΔ*H*, kcal/mol
vardenafil	100:1	−0.1	0.4	0	−0.1
	50:1	−0.2	0.5	0	−0.2
	25:1	−0.3	0.7	0.1	−0.6
	10:1	−0.3	0.8	0.2	−0.7
	5:1	−0.1	0.9	0.3	−0.9
sildenafil	100:1	−0.1	0.1	0	0
	50:1	−0.2	0.2	0	−0.1
	25:1	−0.3	0.2	0	−0.2
	10:1	−0.3	0.3	0	−0.2
	5:1	−0.2	0.3	0.1	−0.3
tadalafil	100:1	−0.1	0.2	0	−0.1
	50:1	−0.1	0.3	0.1	−0.2
	25:1	−0.2	0.3	0.1	−0.3
	10:1	−0.2	0.3	0.2	−0.3
	5:1	−0.2	0.4	0.2	−0.4

Δ*T*_m_, ΔΔ*T_b_*—the changes in the melting temperature of DPPC and the width of the main transition peak. The *T_m_* and Δ*T_b_* of agent-untreated DPPC was equal to 41.5 ± 0.2 and 1.7 ± 0.1 °C, respectively; ΔΔ*T_h_*—the changes in the difference in the transition temperatures between heating and cooling scans (alteration in *T_m_*-hysteresis); ΔΔ*H*—the changes in the transition enthalpy. In the absence of agents, Δ*T_h_* and Δ*H* was equal to 0.4 ± 0.1 °C and 25.0 ± 0.5 kcal/mol, respectively.

**Table 2:** PDE inhibitors influence on the properties of membranes composed of POPI/POPS/POPC/POPE/SSM/cholesterol (6/12/46/32/22/82) assessed by MD simulation at 37 °C. Lipid:drug molar ratio was 10:1.

	Area per Lipid, Å^2^	Thickness, Å
control	42.42 ± 0.39	46.42 ± 0.40
vardenafil	46.64 ± 0.83	44.07 ± 0.55
sildenafil	48.48 ± 0.75	44.22 ± 0.51
tadalafil	44.72 ± 0.69	45.01 ± 0.54

## Data Availability

No new data were created or analyzed in this study. Data sharing is not applicable to this article.

## Supplementary Materials

The following supporting information can be downloaded at: https://www.mdpi.com/article/10.3390/pharmaceutics17050563/s1, Figure S1. Root mean square deviation (RMSD) values of the membrane coordinates of (a) DMPC, DPPC, and DSPC membranes and (b) POPI/POPS/POPC/POPE/SSM/cholesterol (6/12/46/32/22/82 mol%) bilayers in the absence and presence of agent.

## Abbreviations

The following abbreviations are used in this manuscript:

PDE5	Phosphodiesterase 5
cGMP	Guanosine monophosphate
DMPC	1,2-dimyristoyl-*sn*-glycero-3-phosphocholine
DPPC	1,2-dipalmitoyl-*sn*-glycero-3-phosphocholine
DSPC	1,2-distearoyl-*sn*-glycero-3-phosphocholine
DAPC	1,2-diarachidoyl-*sn*-glycero-3-phosphocholine
POPI	1-palmitoyl-2-oleoyl-inositol
POPS	3-palmitoyl-2-oleoyl- D-glycero-1-phosphatidylserine
POPC	3-palmitoyl-2-oleoyl-D-glycero-1-phosphatidylcholine
POPE	3-palmitoyl-2-oleoyl-D-glycero-1-phosphatidylethanolamine
SSM	sphingomyelin

## References

[1] Andersson K.E. (2018). PDE5 inhibitors—Pharmacology and clinical applications 20 years after sildenafil discovery. Br. J. Pharmacol..

[2] Samidurai A., Xi L., Das A., Kukreja R.C. (2023). Beyond Erectile Dysfunction: CGMP-Specific Phosphodiesterase 5 Inhibitors for Other Clinical Disorders. Annu. Rev. Pharmacol. Toxicol..

[3] Luo W., Liu R., Cai X., Zhou Q., Zhang C. (2025). Molecular Dynamics-Assisted Discovery of Novel Phosphodiesterase-5 Inhibitors Targeting a Unique Allosteric Pocket. Molecules.

[4] Ganapathy A.A., Hari Priya V.M., Baby K., Bindhu S., Jayan R., Krishnamoorthi R., Somappa S.B., Nayak Y., Kumaran A. (2024). Flavone-C-glycosides from *Cassia auriculata* L. as possible inhibitors of phosphodiesterase-5 (PDE5): In vitro, molecular docking and molecular dynamics studies. J. Biomol. Struct. Dyn..

[5] Dash P., Bala Divya M., Guruprasad L., Guruprasad K. (2018). Three-dimensional models of Mycobacterium tuberculosis proteins Rv1555, Rv1554 and their docking analyses with sildenafil, tadalafil, vardenafil drugs, suggest interference with quinol binding likely to affect protein’s function. BMC Struct. Biol..

[6] Black K.L., Yin D., Ong J.M., Hu J., Konda B.M., Wang X., Ko M.K., Bayan J.A., Sacapano M.R., Espinoza A. (2008). PDE5 inhibitors enhance tumor permeability and efficacy of chemotherapy in a rat brain tumor model. Brain Res..

[7] Li Q., Shu Y. (2014). Pharmacological modulation of cytotoxicity and cellular uptake of anti-cancer drugs by PDE5 inhibitors in lung cancer cells. Pharm. Res..

[8] Wang R., Chen W., Zhang Q., Liu Y., Qiao X., Meng K., Mao Y. (2015). Phosphodiesterase type 5 inhibitor Tadalafil increases Rituximab treatment efficacy in a mouse brain lymphoma model. J. Neurooncol..

[9] Hu J., Ljubimova J.Y., Inoue S., Konda B., Patil R., Ding H., Espinoza A., Wawrowsky K.A., Patil C., Ljubimov A.V. (2010). Phosphodiesterase type 5 inhibitors increase Herceptin transport and treatment efficacy in mouse metastatic brain tumor models. PLoS ONE.

[10] Sinha B., Köster D., Ruez R., Gonnord P., Bastiani M., Abankwa D., Stan R.V., Butler-Browne G., Vedie B., Johannes L. (2011). Cells respond to mechanical stress by rapid disassembly of caveolae. Cell.

[11] Parton R.G., del Pozo M.A. (2013). Caveolae as plasma membrane sensors, protectors and organizers. Nat. Rev. Mol. Cell Biol..

[12] Zakharova A.A., Efimova S.S., Ostroumova O.S. (2021). Phosphodiesterase Type 5 Inhibitors Greatly Affect Physicochemical Properties of Model Lipid Membranes. Membranes.

[13] DrugBank. https://go.drugbank.com.

[14] Vanommeslaeghe K., MacKerell A.D. (2012). Automation of the CHARMM General Force Field (CGenFF) I: Bond perception and atom typing. J. Chem. Inf. Model..

[15] Mishra S., Meuwly M. (2009). Nitric oxide dynamics in truncated hemoglobin: Docking sites, migration pathways, and vibrational spectroscopy from molecular dynamics simulations. Biophys. J..

[16] Abraham M.J., Murtola T., Schulz R., Páll S., Smith J.C., Hess B., Lindahl E. (2015). GROMACS: High performance molecular simulations through multi-level parallelism from laptops to supercomputers. SoftwareX.

[17] Lee J., Cheng X., Swails J.M., Yeom M.S., Eastman P.K., Lemkul J.A., Wei S., Buckner J., Jeong J.C., Qi Y. (2016). CHARMM-GUI Input Generator for NAMD, GROMACS, AMBER, OpenMM, and CHARMM/OpenMM Simulations Using the CHARMM36 Additive Force Field. J. Chem. Theory Comput..

[18] Jo S., Lim J.B., Klauda J.B., Im W. (2009). CHARMM-GUI Membrane Builder for mixed bilayers and its application to yeast membranes. Biophys. J..

[19] Baron C.B., Coburn R.F. (2004). Smooth muscle raft-like membranes. J. Lipid Res..

[20] Bussi G., Donadio D., Parrinello M. (2007). Canonical sampling through velocity rescaling. J. Chem. Phys..

[21] Bernetti M., Bussi G. (2020). Pressure control using stochastic cell rescaling. J. Chem. Phys..

[22] Darden T., York D., Pedersen L. (1993). Particle mesh Ewald: An N⋅log(N) method for Ewald sums in large systems. J. Chem. Phys..

[23] Humphrey W., Dalke A., Schulten K. (1996). VMD: Visual molecular dynamics. J. Mol. Graph..

[24] Guixà-González R., Rodriguez-Espigares I., Ramírez-Anguita J.M., Carrió-Gaspar P., Martinez-Seara H., Giorgino T., Selent J. (2014). MEMBPLUGIN: Studying membrane complexity in VMD. Bioinformatics.

[25] Yuan H., Jameson C.J., Murad S. (2009). Exploring gas permeability of lipid membranes using coarse-grained molecular dynamics. Mol. Simul..

[26] Slater J.L., Huang C.H. (1988). Interdigitated bilayer membranes. Prog. Lipid Res..

[27] Lu T., Guo T. (2018). Phase Behavior of Lipid Bilayers: A Dissipative Particle Dynamics Simulation Study. Adv. Theory Simul..

[28] Mavromoustakos T., Chatzigeorgiou P., Koukoulitsa C., Durdagi S. (2010). Partial interdigitation of lipid bilayers. Int. J. Quantum Chem..

[29] Smith E.A., Dea P.K. (2013). Differential Scanning Calorimetry Studies of Phospholipid Membranes: The Interdigitated Gel Phase. Applications of Calorimetry in a Wide Context—Differential Scanning Calorimetry, Isothermal Titration Calorimetry and Microcalorimetry.

[30] Chen J.J., Sun Y.L., Tiwari A.K., Xiao Z.J., Sodani K., Yang D.H., Vispute S.G., Jiang W.Q., Chen S.D., Chen Z.S. (2012). PDE5 inhibitors, sildenafil and vardenafil, reverse multidrug resistance by inhibiting the efflux function of multidrug resistance protein 7 (ATP-binding Cassette C10) transporter. Cancer Sci..

[31] Ding P.R., Tiwari A.K., Ohnuma S., Lee J.W., An X., Dai C.L., Lu Q.S., Singh S., Yang D.H., Talele T.T. (2011). The phosphodiesterase-5 inhibitor vardenafil is a potent inhibitor of ABCB1/P-glycoprotein transporter. PLoS ONE.

[32] Möller M.N., Denicola A. (2018). Diffusion of nitric oxide and oxygen in lipoproteins and membranes studied by pyrene fluorescence quenching. Free Radic. Biol. Med..

[33] Denicola A., Souza J.M., Radi R., Lissi E. (1996). Nitric oxide diffusion in membranes determined by fluorescence quenching. Arch. Biochem. Biophys..

[34] Subczynski W.K., Lomnicka M., Hyde J.S. (1996). Permeability of nitric oxide through lipid bilayer membranes. Free Radic. Res..

[35] Mamonov A.A., Stefanov V.E., Shchegolev B.F. (2009). Molecular dynamics investigation of nitric oxide (II) interaction with a model biological membrane. Biochem. Moscow Suppl. Ser. A.

