# Supplementary material for: Membrane-Mediated Action of Phosphodiesterase 5 Inhibitors
Source: Pharmaceutics. 2025 Apr 24;17(5):563. doi: 10.3390/pharmaceutics17050563 (PMC12115186; doi:10.3390/pharmaceutics17050563)
Supplement: Supplementary file 1 [file pharmaceutics-17-00563-s001.zip › pharmaceutics-3559329-supplementary.pdf]

SUPPLEMENTARY MATERIALS

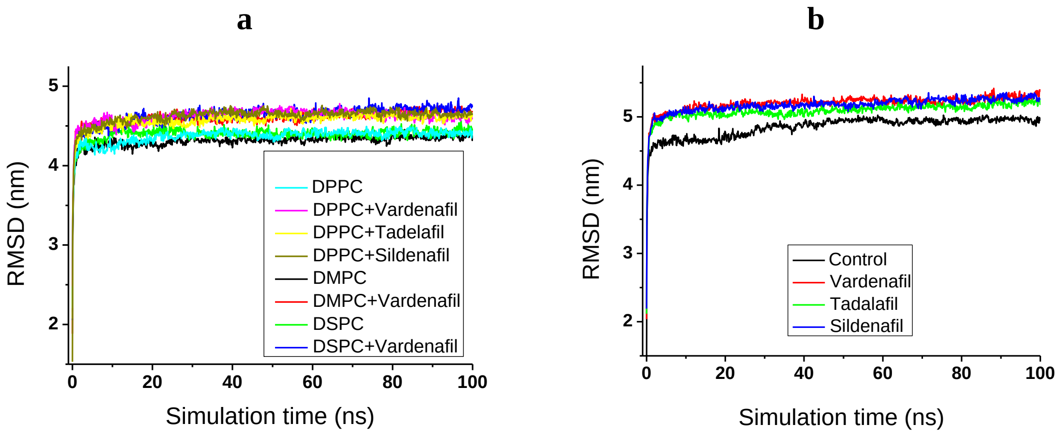

Figure S1. Root mean square deviation (RMSD) values of the membrane coordinates of (**a**) DMPC, DPPC, and DSPC membranes and (b) POPI/POPS/POPC/POPE/SSM/cholesterol (6/12/46/32/22/82 mol%) bilayers in the absence and presence of agent.
